# Supplementary material for: Incorporation of NiO into SiO2, TiO2, Al2O3, and Na4.2Ca2.8(Si6O18) Matrices: Medium Effect on the Optical Properties and Catalytic Degradation of Methylene Blue
Source: Nanomaterials (Basel). 2020 Dec 10;10(12):2470. doi: 10.3390/nano10122470 (PMC7763803; doi:10.3390/nano10122470)
Supplement: Supplementary file 1 [file nanomaterials-10-02470-s001.pdf]

nanomaterials

Supporting Information

Incorporation of NiO into SiO_2, TiO_2, Al_2O_3, and $Na_{4.2}Ca_{2.8}(Si_6O_{18})$ Matrices: Medium Effect on the Optical Properties and Catalytic Degradation of Methylene Blue

Carlos Diaz [1,*], Maria L. Valenzuela [2], Olga. Cifuentes-Vaca [3], Marjorie Segovia [1] and Miguel A. Laguna-Bercero [4,*]

1 Departamento de Química, Facultad de Química, Universidad de Chile, La Palmeras 3425, Nuñoa, casilla 653, post code 7800003, Santiago de Chile, Chile; msmonrroy@gmail.com
2 Facultad de Ingeniería, Instituto de Ciencias Químicas Aplicadas, Inorganic Chemistry and Molecular Material Center, Universidad Autónoma de Chile, Av. El Llano Subercaseaux 2801, San Miguel, 8910060 Santiago de Chile, Chile
3 Departamento Ciencias Químicas, Facultad de Ciencias Exactas, Universidad Andres Bello, sede Concepción, Autopista Concepción-Talcahuano 7100 Talcahuano, Chile; olcifuen@hotmail.com
4 Instituto de Nanociencia y Materiales de Aragón (INMA), CSIC-Universidad de Zaragoza, 50009 Zaragoza, Spain; malaguna@unizar.es
* Correspondence: cdiaz@uchile.cl (C.D.); malaguna@unizar.es (M.A.L.B.)

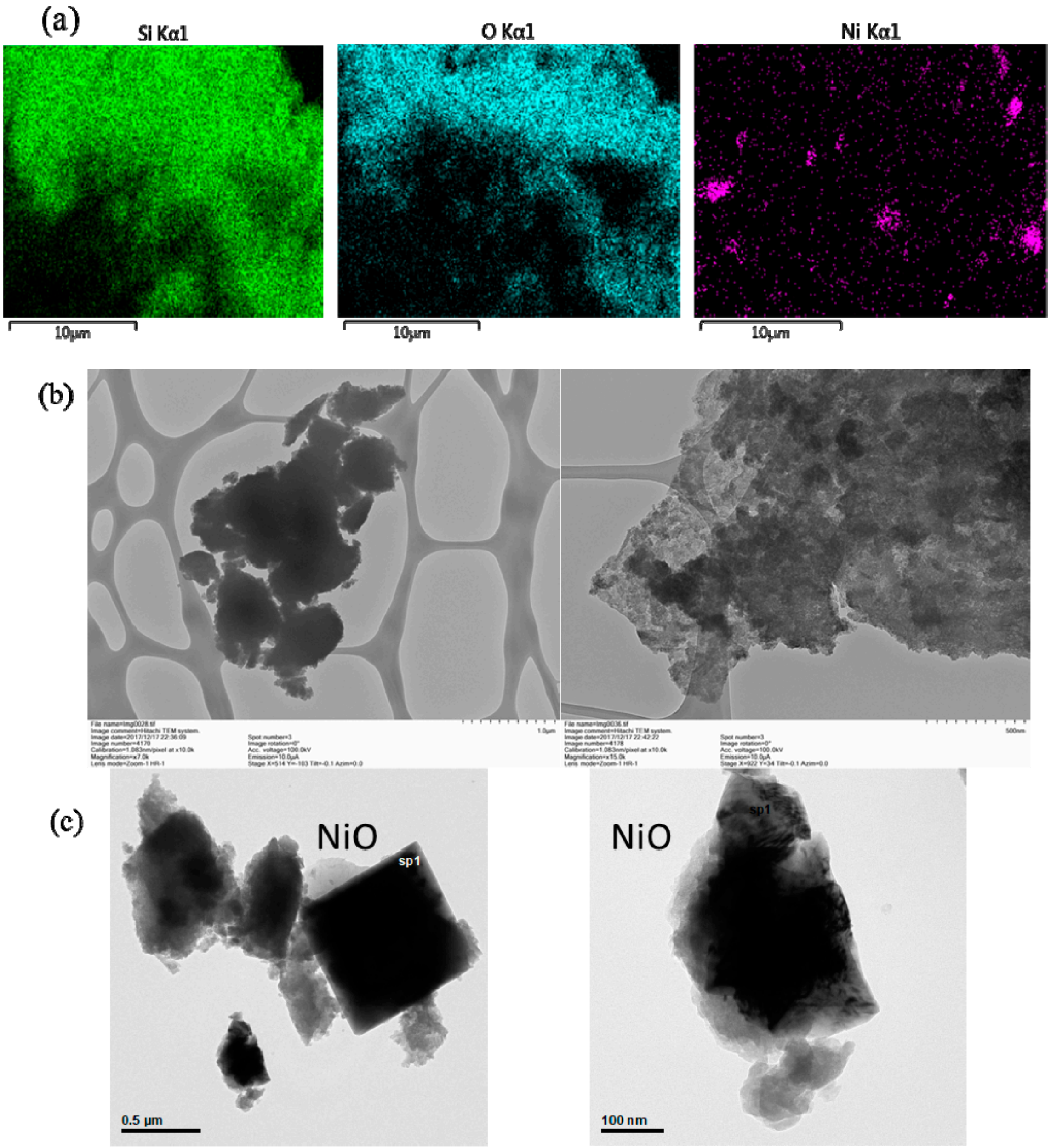

Figure S1. (**a**) SEM-EDS mapping by element and (**b**) TEM images and (**c**) HRTEM image of NiO from the PS-co-4-PVP·($NiCl_2$)x //SiO_2 precursor.

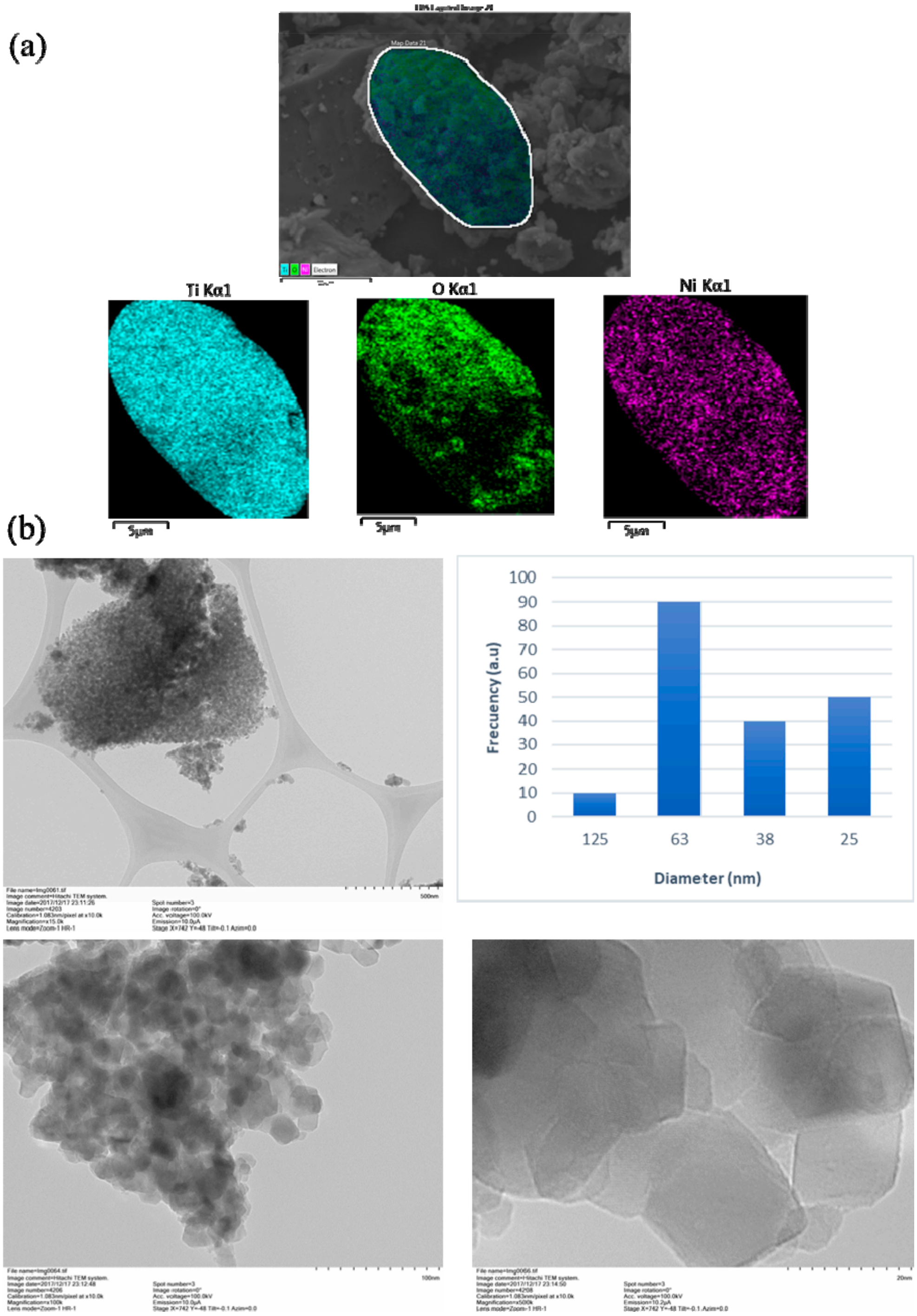

Figure S2. (**a**) SEM-EDS mapping by element from the PS-co-4-PVP·$(NiCl_2)n)x//TiO_2$ precursor and (**b**) TEM images and its histogram of the pyrolytic product from the PS-co-4-PVP·($NiCl_2)n)x//TiO_2$ precursor.

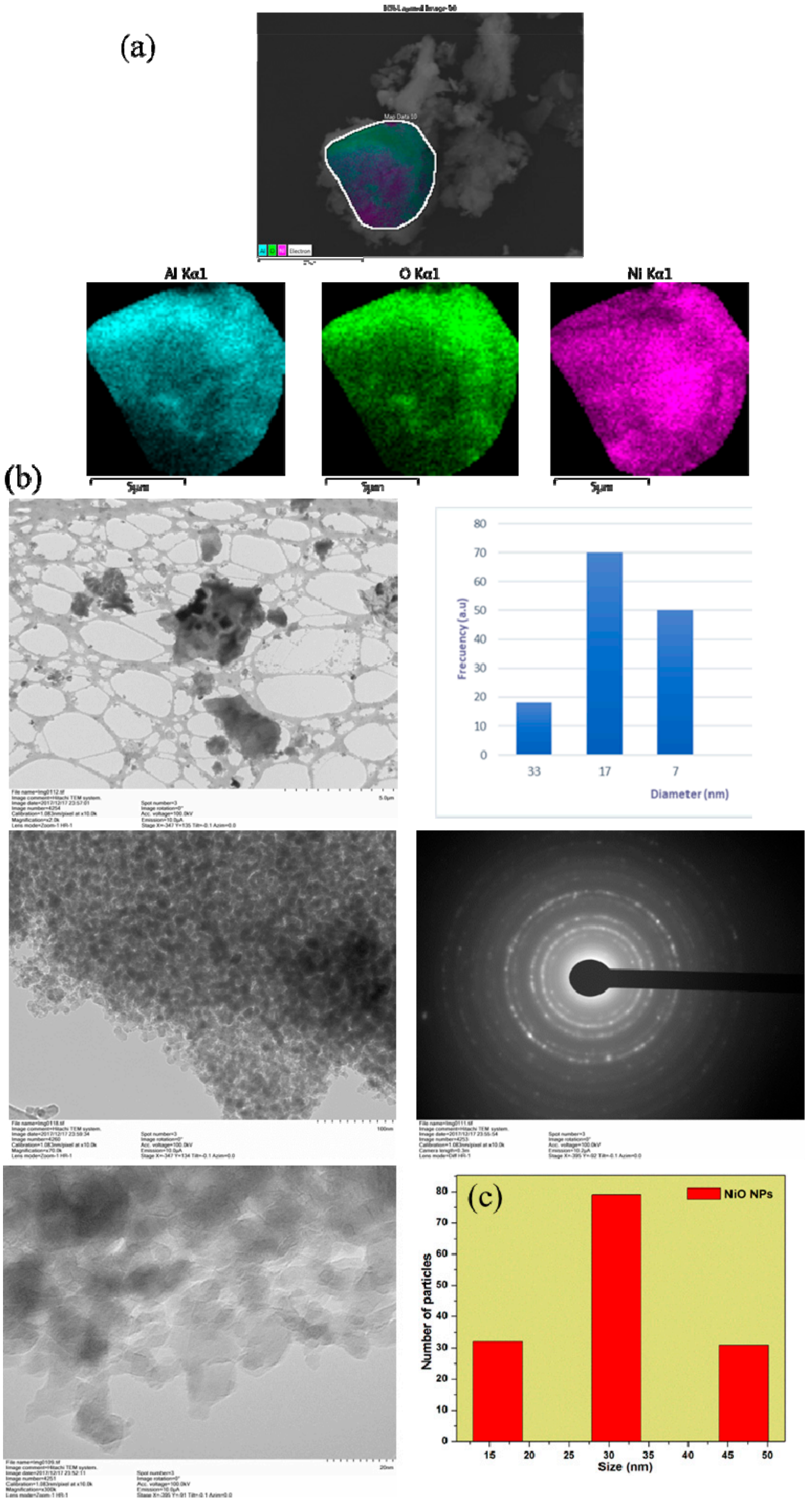

Figure S3. (**a**) SEM-EDS mapping by element and (**b**) TEM images, Histogram and electron diffractions, of the pyrolytic product from the precursor PS-co-4-PVP·$(NiCl_2)n)x$ //Al_2O_3 and (**c**) Histogram of TEM image of NiO from Chitosan·$(NiCl_2)n)x$ //Al_2O_3 precursor .

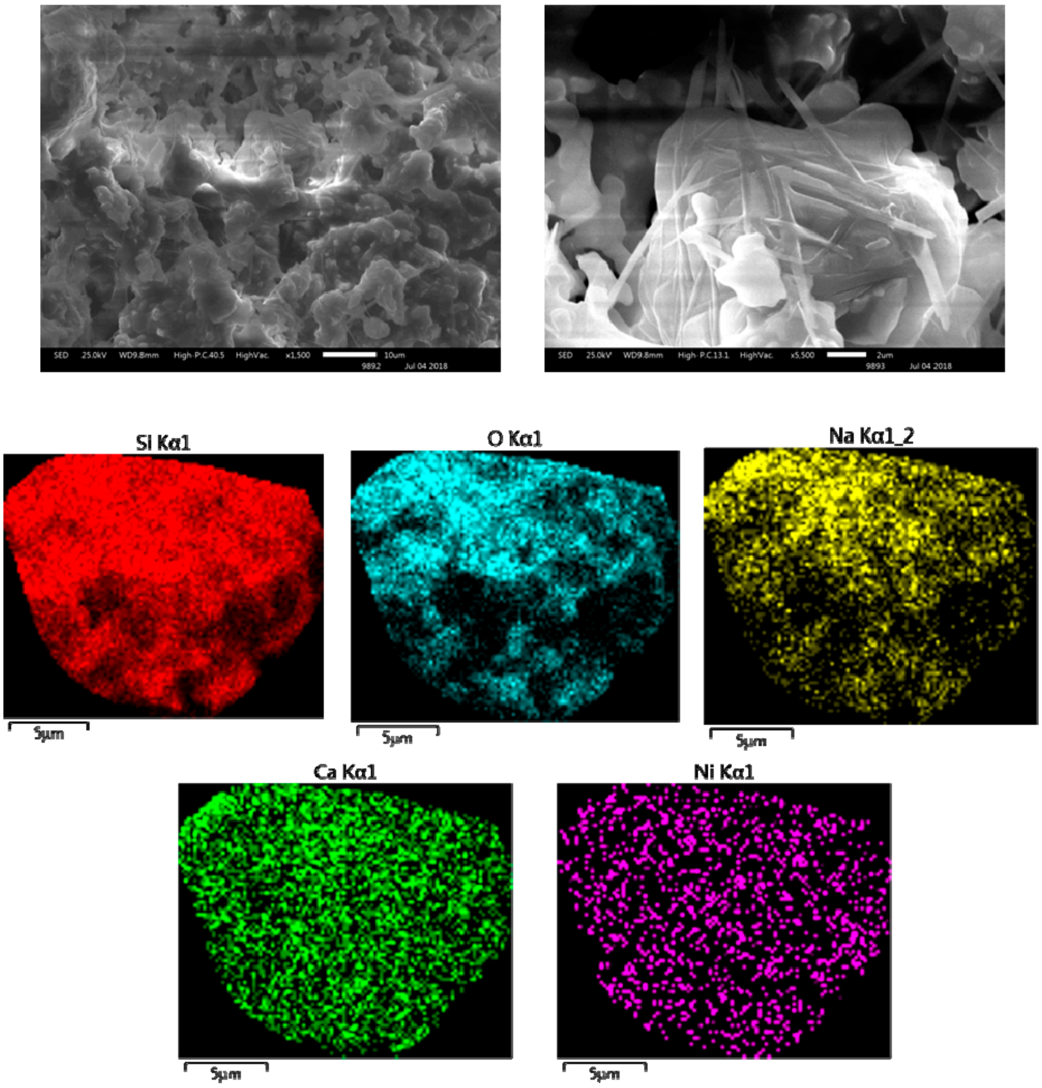

Figure S4. SEM image and EDS mapping analysis for the composite NiO/$Na_{4.2}Ca_{2.8}(Si_6O_{18})$ from the PS-co-4-PVP·$(NiCl_2)n$)//SiO_2·CaO·Na_2O precursor.

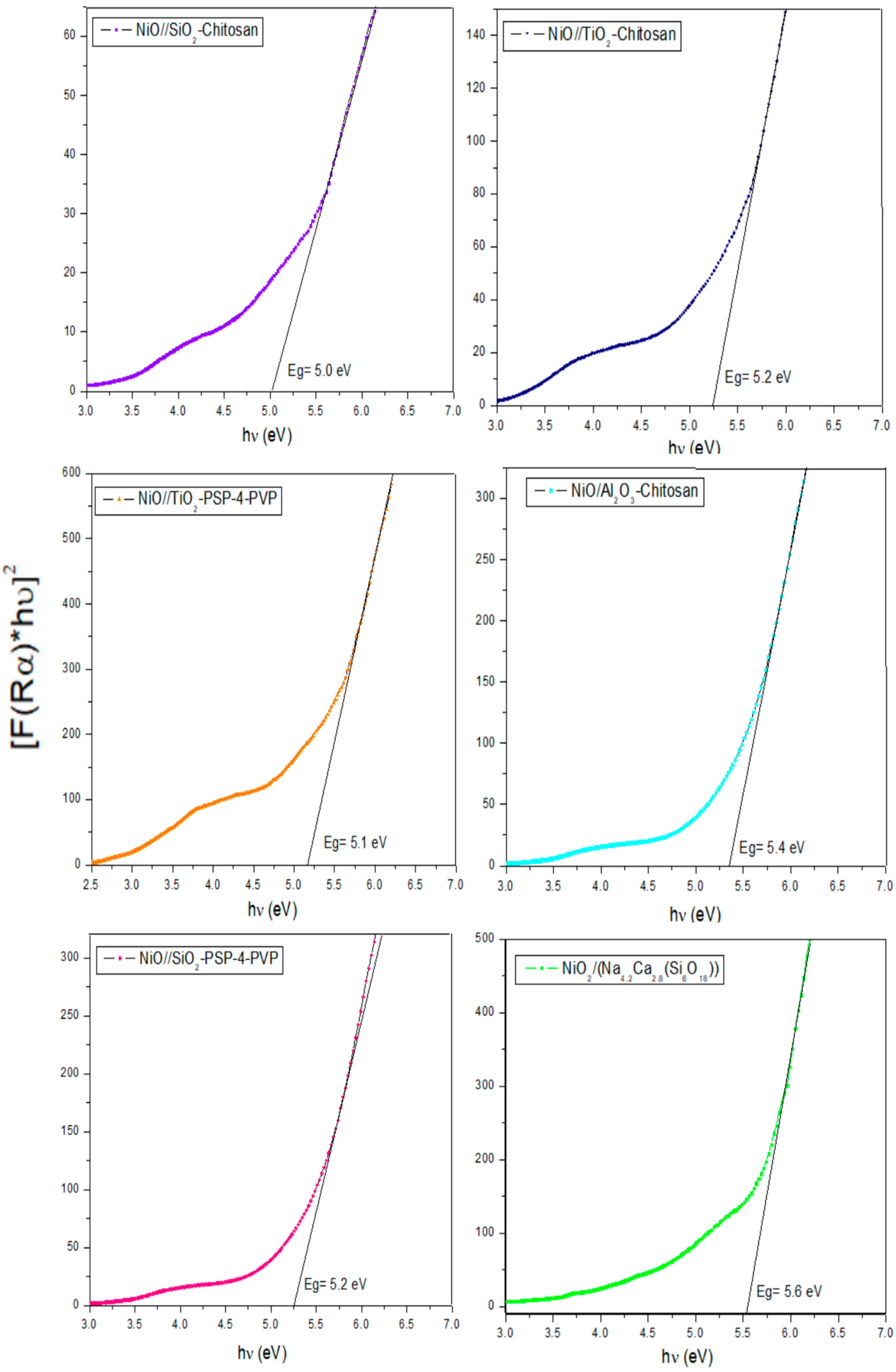

Figure S5. Tauc determination of Eg values for NiO from Chitosan·$(NiCl_2)n$. and PS-co-4-PVP·$(NiCl_2)n$ and for NiO/SiO_2, NiO/TiO_2, NiO/Al_2O_3 and $NiO/Na_{4.2}$ $Ca_{2.8}(Si_6$ $O_{18})$ composites.

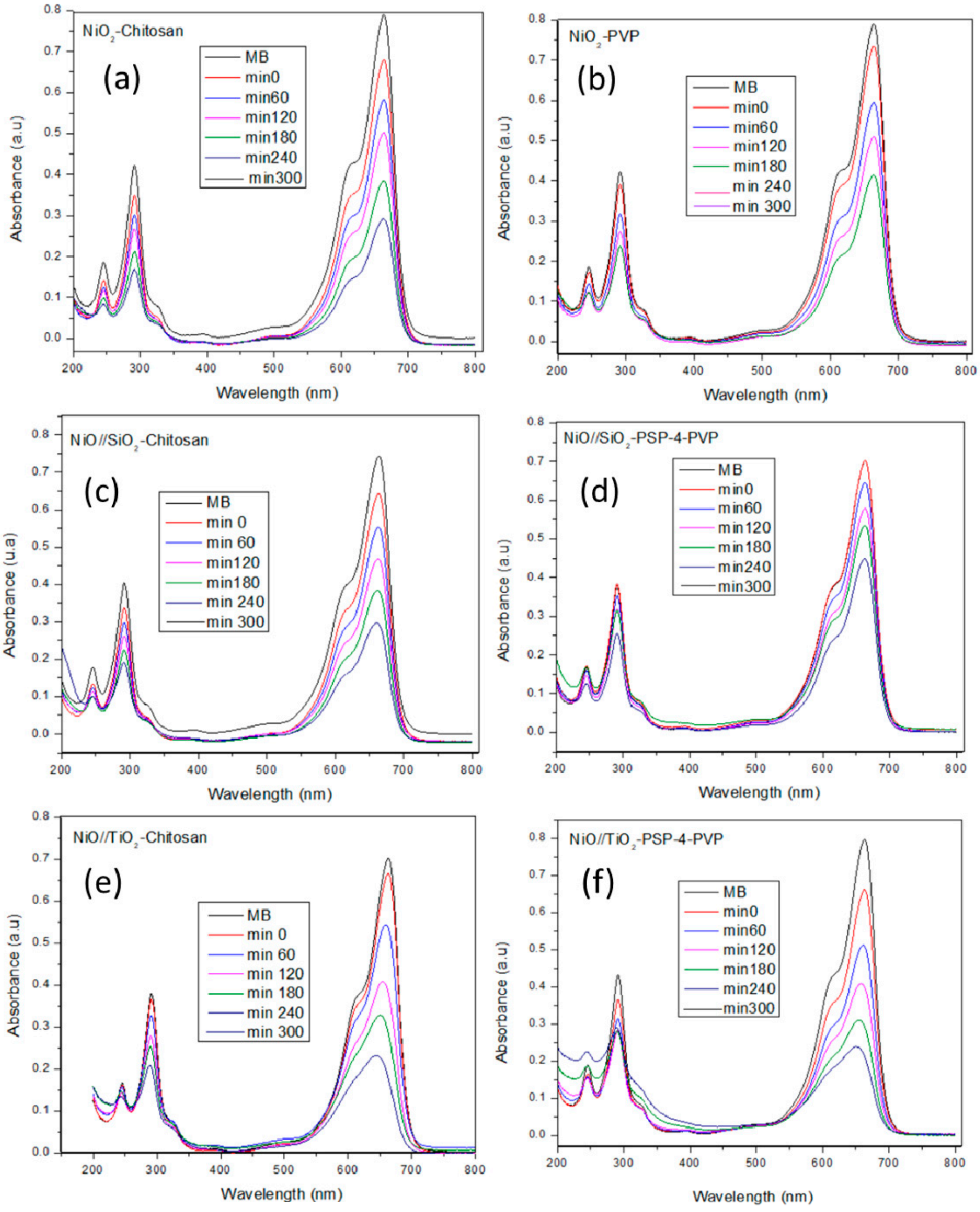

(a)
NiO$_2$-Chitosan
MB
min0
min60
min120
min180
min240
min300
(b)
NiO$_2$-PVP
MB
min0
min60
min120
min180
min 240
min 300
(c)
NiO//SiO$_2$-Chitosan
MB
min 0
min 60
min120
min180
min 240
min 300
(d)
NiO//SiO$_2$-PSP-4-PVP
MB
min0
min60
min120
min180
min240
min300
(e)
NiO//TiO$_2$-Chitosan
MB
min 0
min 60
min 120
min 180
min 240
min 300
(f)
NiO//TiO$_2$-PSP-4-PVP
MB
min0
min60
min120
min180
min240
min300
Absorbance (a.u)
Absorbance (u.a)
Wavelength (nm)

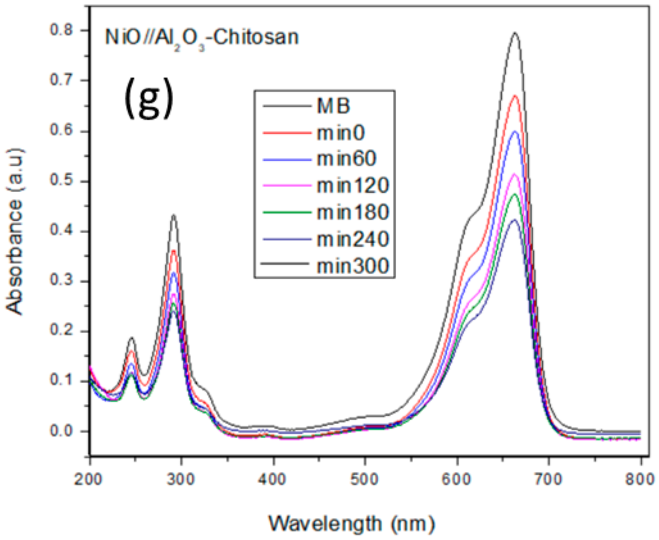

Figure S6. Absorbance vs time for the blue methylene degradation for NiO (a) and for the composites NiO/SiO_2, NiO/TiO_2, NiO/Al_2O_3; (**b**) NiO/SiO_2 from Chitosan·($NiCl_2$)x//TiO_2; (**c**) NiO/SiO_2 from PS-co-4-PVP·($NiCl_2$)x//TiO_2; (**d**) NiO/TiO_2 from Chitosan·($NiCl_2$)x //TiO_2; (**e**) NiO/TiO_2 from PS-co-4-PVP·($NiCl_2$)x //TiO_2; (**f**) NiO/Al_2O_3 from Chitosan ($NiCl_2$)x //Al_2O_3; (**g**) NiO/ $Na_{4.2}Ca_{2.8}(Si_6O_{18})$ from Chitosan·($NiCl_2$)x // Na_2O·CaO·SiO_2

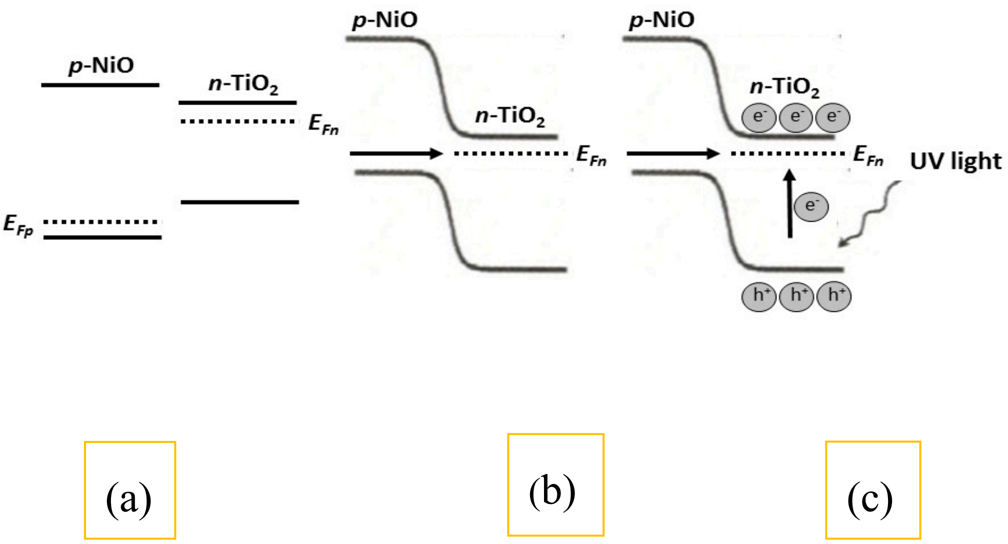

Figure S7. Schematic diagrams for (**a**) energy bands of p-NiO and TiO_2 before contact, (**b**) formation of p-n junction and its energy diagram at equilibrium and (**c**) transfer of holes from n-TiO2 to p-NiO under UV irradiation.

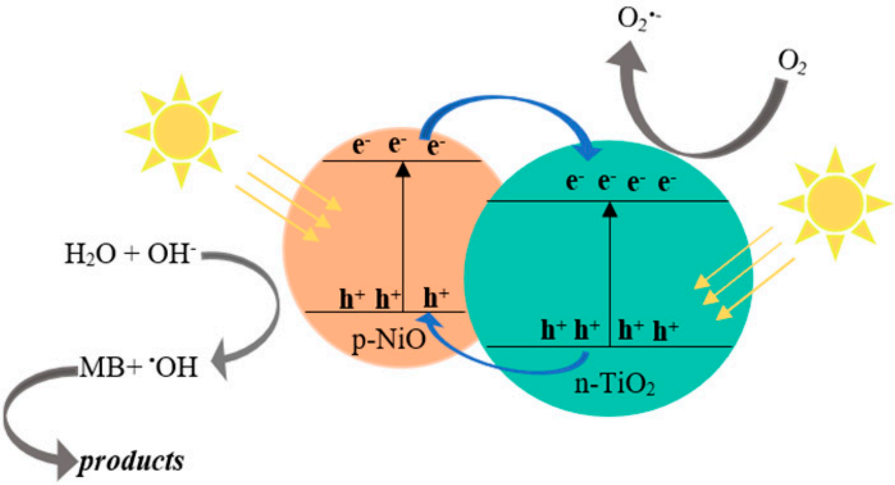

Figure S8. The photodegradation mechanism of NiO/TiO2 composites.

When NiO-TiO_2 nanocomposites are illuminated by visible light, the electrons of the Ni 3d sub-band are excited and transferred to the conduction band (see figure S11 in supplementary information). Accordingly, a high flux of free electrons is produced in the conduction band of TiO_2. The photogenerated electrons in the conduction band of TiO_2 reduce O_2 species to O^{2-}. This pathway is crucial to promote the photocatalytic efficiency in the MB oxidative decomposition. Then, the photoinduced holes (h^+) in the valence bond of TiO_2 are moved to the valence bond of NiO, generating a high flow of holes at the NiO interface. These holes react with H_2O or OH^- ions, producing extremely oxidative OH radicals, which proper oxidants in the photocatalytic oxidation process. These radicals react rapidly with methylene blue.

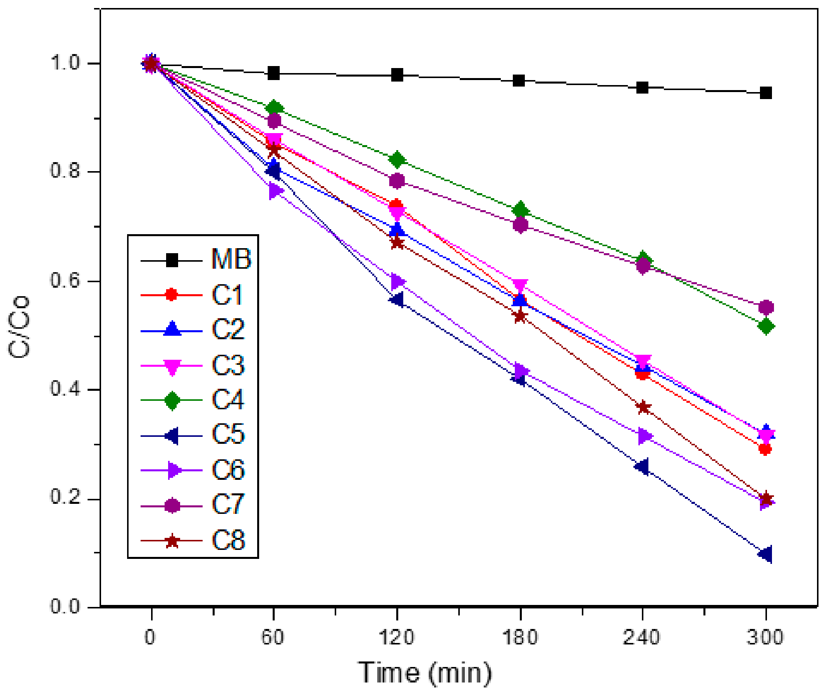

Figure S9. Kinetic plot of the blue methylene degradation with the composites NiO/SiO_2, NiO/TiO_2, NiO/Al_2O_3 and NiO/$Na_{4.2}Ca_{2.8}(Si_6O_{18})$.

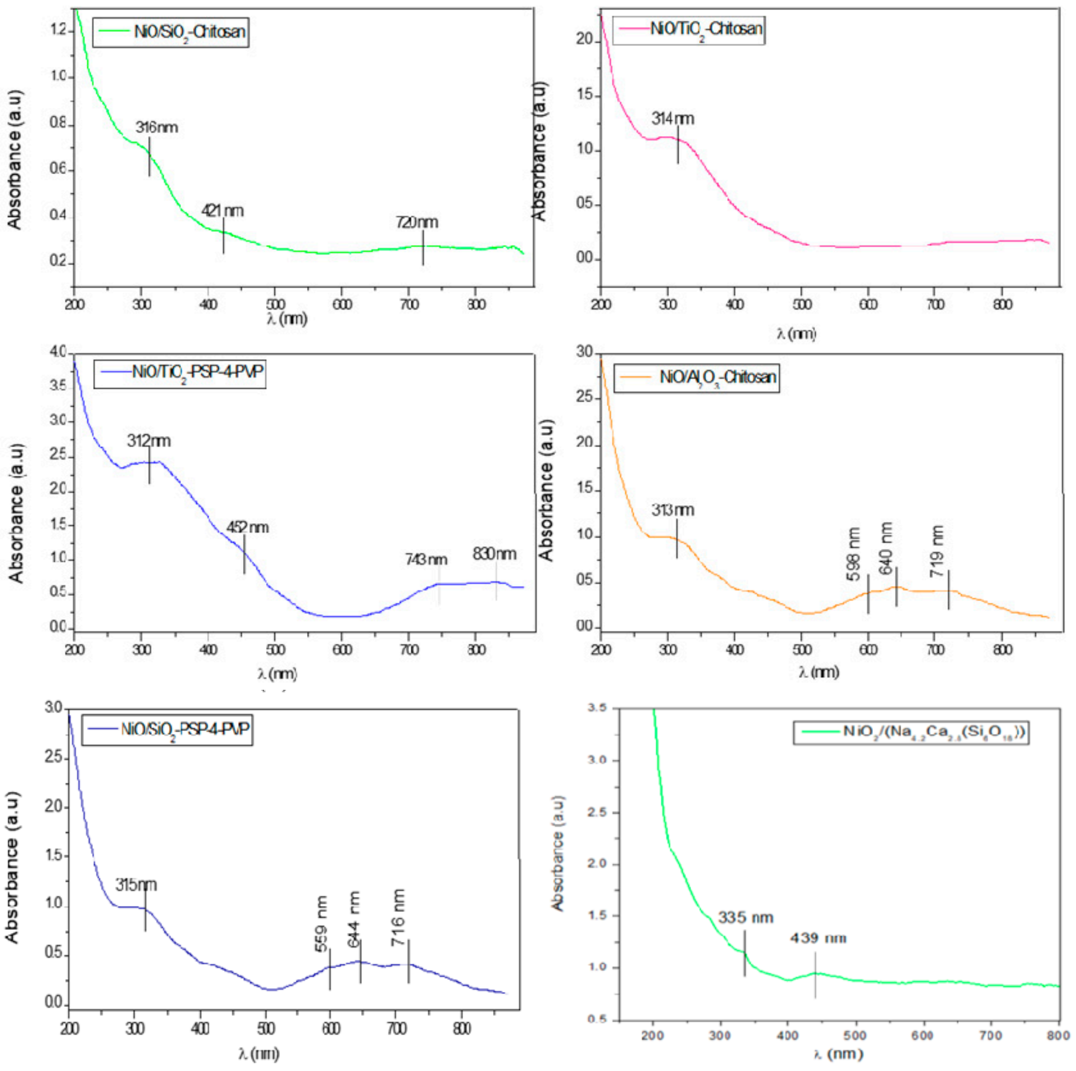

Figure S10. UV-Vis absorption spectra of the composites.

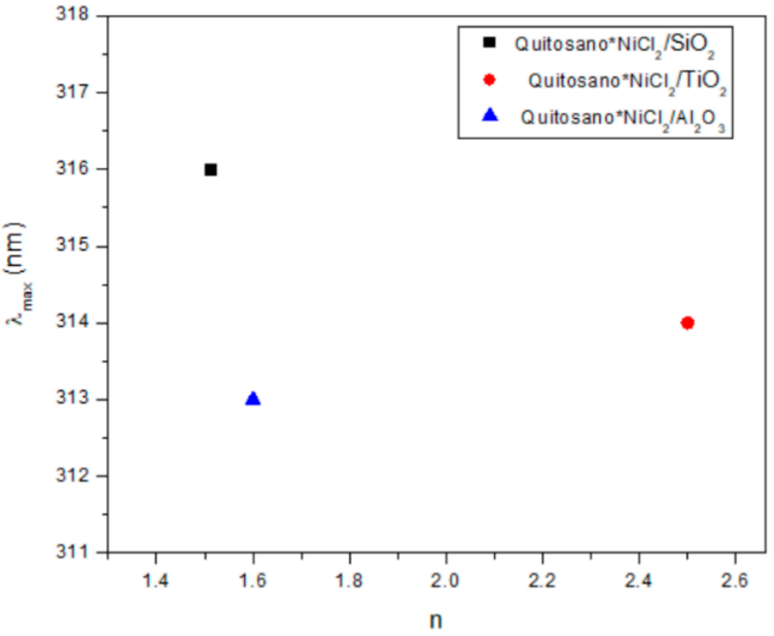

Figure S11. Plot of λ_{max} for the NiO vs the refractive index for the matrices SiO_2, TiO_2 and Al_2O_3.
